# East and West

**Published:** 1887-11-05

**Authors:** Leslie Keith

**Affiliations:** Author of "The Chilcotes," "Uncle Bob's Niece," etc.


### East and West.

By Leslie Keith.

Author of "The Chilcotes," "Uncle Bob's Niece," etc.

(*Continued from p.* 86.)

Chapter VI.

Mrs. Carter began at this time to be a person of great importance, and it was even believed by certain of the ignorant small fry who billeted themselves on the doorsteps that her fame had spread to that unknown quarter of London where the great live, and where the Queen is sometimes to be seen by those privileged folks with her crown on her head and her sceptre in her hand.

For to visit Mrs. Carter there had first of all arrived a gentleman—an undoubted "swell," who came in a hansom. He had tossed money to the cabby on his perch, and the cabby in response had touched his hat, which is never done under a handsome tip; he had walked into the shop, and the children, watching this interesting incident from a safe distance, had witnessed him being ushered into the back parlour. Thence he had in due time come out, Mrs. Carter accompanying him to the door and curtseying to him there—not a mere mutilated bob, but a deep and reverential bend, expressive of the utmost respect.

The gentleman walked away with a half smile on his face, but presently he turned and surveyed the neighbourhood. It was a sunny day, but the light only served to emphasize the shabbiness of Lavender Lane. A good many untidy heads were thrust forth from windows—a public-house opposite the general shop he had quitted was doing a most flourishing trade, and there were the children from the biggest to the smallest in one unanimous stare. When his eyes fell on these last the gentleman put an irresolute hand into his pocket—instantly the battery of glances followed his action. If he had drawn forth his hand empty there might have been a howl of execration, even, possibly, an onslaught of fury. He drew it forth full of small, jingling coins, and there was a shriek of discordant joy.

This exciting incident was, however, swept clean from the public memory by the arrival, a few days later, of a carriage drawn by a dashing pair of greys, and duly adorned with a fat coachman and a tall footman. Within were seen a lady and gentleman, and this, to be sure, brought all the heads to the windows once more, especially as it was evident the lady meant to descend and interview Mrs. Carter. She looked rather frightened as she glanced round her with half shy curiosity, but she had scarcely been gone two minutes when she returned.

"So soon back, Kitty?" said the young man who had waited for her, sauntering up and down the pavement under the fire of public criticism.

"They are not here, Jack. They have gone away—to Baron Street. Have you ever heard of it?"

"Never, but we'll find it. I feel like an explorer, don't you?"

"If to feel horrid is to feel like one. Look at me, Jack."

This was a command the young man was quite ready to obey.

"You manage to conceal your feelings successfully," he said, with due gravity.

She shook her head. "You know what I mean. I am different from them, and they are angry. I ought not to be wearing this dress or riding in this carriage. That is what they were all saying just now. I saw it in their faces. Alicia was right: we ought to have come in an omnibus."

There was a lavish outlay in Lavender Lane on ounces of tea and sugar that night—a Saturday night, as it conveniently happened—but the owner of the tea was "close," and not discursive, and she wore an air that chilled curiosity. Lavender Lane was left to its own conjectures, but it knew that the visit was for the brother and sister from the North—Mrs. Carter's late lodgers. Therefore it did not become Mrs. Carter to be proud, and it was the general opinion that it would be wholesome for her "to be took down."

Mary was not proud, so long as you did not offer her charity, and her feeling on this point she called honest independence. She was startled, surprised, and shy when this vision of slim youth and beauty came into her room. Kitty was shy too, but was comforted by the knowledge of Jack's stalwart person behind the door. The heart under Kitty's pretty bodice was indeed beating at an unusual pace, for her adventure had as yet presented more of novelty than of charm.

The sovereign people is not very polished when you meet it in its own quarters. It smokes bad tobacco in your face; it will not budge an inch out of the path for your greater convenience, and if you should happen to be, as Kitty was, young and pretty, its personal comments do not lack abundant frankness. A dreadful old woman had been encountered on the stair—a Bacchantic old woman with wild locks and staring eyes, who diffused a fiery breath of gin, and who spoke with an audacity that would have put to the blush even the most audacious old lady in society. Kitty had never met her like; perhaps she had mostly thought of the poor as she had read of them in books, a virtuous and happy peasantry with curtseys and smiling looks, and clean, dusted chairs for the gentry.

Mary more nearly approached this ideal, except that she did not curtsey. She put down her work hastily, and rose, flushing all over her pale face.

Kitty took a step forward.

"I am Catherine Frankland," she said, with a pretty, shy dignity that sat well upon her.

"Yes," said Mary, "I knew it must be. Your father told me of you."

"Papa!"

"Yes; he has been to see us twice. He has promised to get David some work to do when—if—"

"David—that is your brother?"

"Yes; he is out." Mary hung her head. "He—he can't bear to stay indoors; he is restless. It is all so different now—" She faltered and ceased.

"Will you tell me about your home? It was in the country, was it not? In Scotland? I have been there more than once."

"Oh, no, no," said Mary; "I can't talk of it." She had taken up her work again, and was sewing fiercely.

Kitty sat in a guilty and embarrassed silence, not knowing how to mend her unhappy phrase, and it was Mary who came to the rescue.

"Forgive me," she said; "it was home to me all my life till three months ago, and I can't talk of it yet. It was so unlike this that it might have belonged to another world. We knew nothing down there; it was beautiful and peaceful; but I think now perhaps it was a selfish life."

"Oh, no," said Kitty impulsively, looking round her with a quick distaste; "it is very ugly here. I am sure it was far nicer there."

Mary smiled rather sadly.

"It is ugly," she said. "I wish it were less ugly, it makes it so much harder for some of them. There is a poor fellow upstairs—listen, you can hear him cough. He is dying. Think how sad it must be to die without having seen anything of the beautiful world. He was born here, and he has lived here, and now he is going away. That is his history."

"Perhaps he doesn't care, if he never knew anything else," said Kitty, with a hint of resistance in her voice.

"There ought to be a very good sort of Heaven for such people," said Mary musingly; "but if they would only make things a little less ugly here, there wouldn't be so much wickedness. It is a very wicked world, and it isn't these poor people who are only to blame," she said, her pity and her scorn rushing out now that she had found a listener; "and yet we punish them, and the others—those of us who don't think, or care, or try in any way to help them—go free. Could you be good, or I, if we had been brought up since we were little children without seeing a beautiful object or hearing a refined word—without, perhaps, so much as a loving look?"

Kitty got up; she was conscious of an edge of hardness which she could not keep out of her words.

"You can pity them—you can afford to pity them—living here among them as you do."

"So much, so deeply, that I would ask you, if you have a moment, sometimes to think of them—to remember the little children."

"Why don't you go home!" cried Kitty, in passionate interruption. "Why don't you go back to that country place where it was peaceful and—and clean? Why should you stay here? You cannot help; you can only be made uncomfortable."

For all answer Mary suddenly held up her hand.

"There is David," she said; "that is his step."

Her face changed as she spoke. The eagerness and animation died out of it, and were replaced by a strange, yearning anxiety, love and pity—some subtle blending of the three.

Kitty looked at her in amaze. She dimly understood that Mary loved this brother, and suffered an anguish in proportion to her love. He came in presently. Mary's quick ear had heard his footfall, even though it had been crossed by the sentinel pace of Jack Fane keeping guard outside.

David's step was no longer confident; it had that strange languor and hopelessness that tell their own tale significantly enough. His face was worn and hollow; he had always been slim and slight of build; but he seemed a mere ghost of his former self. It was scarce possible to believe that three months could have wrought the change, but they were months of bitter mortification and disappointment and wounded pride. The eyes only were unchanged, unless they were brighter, hungrier, more eager than before.

He came in taking no notice of the visitor; looking at her, indeed, as if he did not see her, with an indifference that might have piqued Kitty had not her vanity been for the moment in subjection to her fear. He looked so strange, so wild, so ill, that she shrank back instinctively, dreading she knew not what.

"Are there no letters?" he asked. He went forward to a table strewn with loose papers, and tossed them over feverishly.

Mary's silence was his answer—his daily answer, for the letter that was to herald fame and fortune never came.

"There is another post presently," she said faintly.

He had buried his head in his hands, and he appeared not to hear her. For a moment his belief in himself, that astounding confidence that recognised no defeat, deserted him. He was weary and faint. When he had rested and had refreshed himself with the meal Mary was preparing his courage would reassert itself. If David had lived it is quite possible that, in time, he might have become, if not a great poet, at least a successful one. He had that quality of dogged faith in himself that is a great deal more useful than genius. Blow your own trumpet but persistently enough, and the big, stupid world will take you at your own estimate and make you the hero you claim to be.

He glanced up presently, and said irritably—

"Where is the ink? You do all you can to hinder me. You know I have to write."

Kitty slipped out of the room, and seized Jack by the arm.

"Take me away; take me home," she said.

"What is it, Kitty? Were they not civil to you?"

"Oh, Jack, don't stay to talk," she implored. "Civil? The girl was well enough, though she talked as if she thought it quite wicked of me to live in Park Lane."

"No; she probably only thought it wicked that she does not live there also," interposed Jack.

"I don't know, I think she likes living there, but the young man——" Kitty gave a little shudder. The young man with the shining, angry eyes had frightened her.

"If they hadn't been our relations," she said, when they were being driven rapidly westwards; "but to be our cousins and to live like that! And they are not people we can help."

"I must say I shouldn't have much cared to offer half-a-crown to the young boss," said Jack, whose ideas of help were rudimentary.

"You couldn't help the girl either. She was sewing at some dreadful stuff that had roughened her fingers, but she is a lady. You can see that. I don't mean that she is one of us." Kitty went on, gropingly explaining her meaning. "I dare say she would despise us, but she is not like the other people with whom she lives. I suppose it isn't, after all, what you've got so much as what you are that makes the difference."

"It must be beastly living down there if you're not used to it," said Jack with conviction.

"Park Lane is certainly nicer, even if it is wicked to live in it," said Kitty with a laugh, quickly followed by a sigh. They were now emerging from the Bethnal Green Road into the bustle of Bishopsgate Street, where the evidences of poverty and want are less apparent; but the sadness of the region she had left went with Kitty. It is always so with those who see it for a first time; there are some, indeed, who, having once seen it, are impelled to face it again to know more of it; there are a few, a very few, who go and do not return, but remain to fight the powers of darkness. Kitty was not one of these; at the most her day's adventure would haunt her and recur like an ugly dream, the impression fainter with every memory of it, until it faded away before some newer and more vivid experience. A dance, a new costume, a gift from the attentive Jack, would serve to rekindle the spark of Kitty's gaiety, eclipsed for the moment.

"Jack," she said, after a silence which the young man spent in wishing he might smoke—"Jack, is it so dreadful to want to be a poet and not to be able to get anybody to listen to you?"

"I never was a poet," said Jack superfluously.

"No," said Kitty pensively, "you never so much as made a sonnet to my eyebrow."

This was undoubtedly true, and the young man bore the reproach with good humour. He had scarcely enough of inspiration, indeed, to save him from being commonplace; but that did not prevent him from remarking that it must be rather a sell to set up to be an immortal and to find nobody to believe in you.

"But it isn't worth dying for!" said Kitty, with a sudden burst of energy.

"No," he acquiesced; "there are few things worth such a big price as that."

Kitty said no more till they had almost reached home; then she sat up and said with a charming smile: "Jack, I haven't told you the queerest thing of all. Think of papa going to see them—twice!"

It was with this astounding news that, as we have seen, she burst in on Alicia. By degrees she poured out the rest of her tale, and possibly the background against which she set it lent it some new force. The contrast between the upper room in Baron Street and the drawing-room in Park Lane could not have been completer if it had been made on purpose to emphasise a moral. Alicia had inherited a fine taste, and she had spent some of that superfluous energy that devoured her in making her father's home the envy of less gifted people. She was always careful to dress so as to be in harmony with her surroundings, and she presented a very pleasing picture to one who had not come straight from Bethnal Green.

Kitty, however, was conscious of a lack of fervour on her sister's part. Alicia listened in a silence that was chilling—her eyes fixed on her book.

The narrator was thus piqued into saying, with warmth:

"I think our cousin David is dying. I don't believe he can live many days. He is either dying, or," she amended, "he is going mad. He looks dreadful."

Then Alicia got up suddenly, her dark eyes were blazing, her cheeks flushed.

"Why do you come and tell me this?" she demanded. "What is it to me? Did I invite him to come to London? Can I help it that he was a weak fool, and believed himself to be a genius for whom the world was waiting? It is you who have chosen to go and see him, not I. It is you who have sought him out. Why do you trouble me about him? If he starves, if he dies, what is that to me?"

She picked up her book, which had fallen, and passing her sister, she left the room with a firm step and a high-held head. Kitty, too shaken to speak, gazed after her in wide-eyed wonder at the storm she had evoked.

*(To be continued.)*

